# Detecting acute distress and risk of future psychological morbidity in critically ill patients: validation of the intensive care psychological assessment tool

**DOI:** 10.1186/s13054-014-0519-8

**Published:** 2014-09-24

**Authors:** Dorothy M Wade, Matthew Hankins, Deborah A Smyth, Elijah E Rhone, Michael G Mythen, David CJ Howell, John A Weinman

**Affiliations:** Chartered Health Psychologist, Critical Care Department, University College Hospital, 235 Euston Rd, London, NW1 2BU UK; Centre for Innovation and Leadership in Health Sciences, Faculty of Health Sciences, University of Southampton, Southampton, SO17 1BJ UK; Critical Care Department, University College Hospital, 235 Euston Rd, London, NW1 2BU UK; School of Medicine, Henriette Raphael House, King’s College London, Guy’s Campus, London, SE1 1UL UK; National Institute of Health Research University College London Hospitals Biomedical Research Centre, Maple House, 149 Tottenham Court Road, London, W1T 7DN UK; Divisional Clinical Director, Critical Care, University College Hospital, 235 Euston Rd, London, NW1 2BU UK; Institute of Pharmaceutical Science, Franklin-Wilkins Building, King’s College London, 150 Stamford Street, London, SE1 9NH UK

## Abstract

**Introduction:**

The psychological impact of critical illness on a patient can be severe, and frequently results in acute distress as well as psychological morbidity after leaving hospital. A UK guideline states that patients should be assessed in critical care units, both for acute distress and risk of future psychological morbidity; but no suitable method for carrying out this assessment exists. The Intensive care psychological assessment tool (IPAT) was developed as a simple, quick screening tool to be used routinely to detect acute distress, and the risk of future psychological morbidity, in critical care units.

**Methods:**

A validation study of IPAT was conducted in the critical care unit of a London hospital. Once un-sedated, orientated and alert, critical care patients were assessed with the IPAT and validated tools for distress, to determine the IPAT’s concurrent validity. Fifty six patients took IPAT again to establish test-retest reliability. Finally, patients completed posttraumatic stress disorder (PTSD), depression and anxiety questionnaires at three months, to determine predictive validity of the IPAT.

**Results:**

One hundred and sixty six patients completed the IPAT, and 106 completed follow-up questionnaires at 3 months. Scale analysis showed IPAT was a reliable 10-item measure of critical care-related psychological distress. Test-retest reliability was good (r =0.8). There was good concurrent validity with measures of anxiety and depression (r =0.7, *P* <0.01; r =0.6, *P* <0.01). With a cut-point of ≥7, the IPAT had 82% sensitivity and 65% specificity to detect concurrent anxiety; and 80% sensitivity and 66% specificity to detect concurrent low mood (area under the curve (AUC) =0.8 for both). Predictive validity for psychological morbidity was good (r =0.4, *P* <0.01; r =0.64, *P* <0.01 for PTSD with days 1 and 2 data). The IPAT had 69% specificity and 57% sensitivity to predict future psychological morbidity (AUC =0.7).

**Conclusions:**

The IPAT was found to have good reliability and validity. Sensitivity and specificity analysis suggest the IPAT could provide a way of allowing staff to assess psychological distress among critical care patients after further replication and validation. Further work is also needed to determine its utility in predicting future psychological morbidity.

**Electronic supplementary material:**

The online version of this article (doi:10.1186/s13054-014-0519-8) contains supplementary material, which is available to authorized users.

## Introduction

There is increasing evidence that the psychological impact of a critical care admission can be severe. Extreme levels of acute distress are common in critically ill patients [1,2], and subsequently there is a high prevalence of psychological morbidity including post-traumatic stress disorder (PTSD), depression and anxiety among critical care survivors [3,4]. Acute distress may be caused by life-threatening illness, real events and medical procedures in critical care, or by frightening psychological experiences such as hallucinations or delusions, that are commonly experienced there [2]. The aetiology of hallucinations and delusions during critical care is not fully understood but they are thought to arise from delirium due to disturbed physiology, infection or inflammation; sleep or sensory deprivation; or the use of, or withdrawal from, psychoactive drugs such as benzodiazepines for sedation.

Acute stress in the critical care unit has also been shown to be one of the strongest risk factors for poor psychological outcomes after critical care [1,2] and therefore, it is important to detect it and minimise it where possible. It is known that healthcare staff who have not been trained in mental health may find it difficult to recognise acute psychological stress, including delirious symptoms, in patients [5,6]. Therefore, many highly distressed patients do not receive psychological support in critical care units, and continue to suffer serious distress after discharge from critical care. In the UK, the 2009 National Institute for Health and Clinical Excellence (NICE) guideline CG83 [7] states that patients should be assessed during their critical care stay for acute symptoms such as anxiety, depression, panic episodes, nightmares, delusions, hallucinations, intrusive memories, flashback episodes and underlying psychological disorders, to determine their risk of future psychological morbidity. Psychological support should be provided as required.

NICE CG83 noted that there was a lack of tools available for detecting acute distress and risk of future psychological morbidity in critically ill patients. A screening tool to detect acute distress in critical care should ideally be administered by bedside nurses to patients. It should be short, to minimise the burden on staff and patients, and simple to understand, so that fatigued, ill patients can respond once they are awake and alert enough to answer questions. It should reflect the many stressful facets of patient experience in critical care including being unable to communicate and sleep, suffering frightening hallucinations and paranoid delusions, as well as symptoms of low mood, panic and anxiety.

Our research team reviewed existing questionnaires for distress in critical care [8-10], but found none were suitable as a quick screening tool. Existing questionnaires were too long, contained many items not relevant to this purpose, or were primarily measures of post-critical care recall rather than current experience of critical care. A recent study reported on a predictive tool to identify patients at risk. However it is based mainly on factors (for example, having children under 18 years of age) that cannot be modified to reduce risk; and informal methods of identifying patient distress [11]. General measures of distress from outside the critical care context, including hospital scales such as the hospital anxiety and depression scale, [12] do not include symptoms such as experience of hallucinations or intrusive memories, which are important sources of distress for critical care patients, and may be key predictors of future psychological morbidity.

PTSD screening tools are not suitable for assessing patients in critical care units as many indicators relevant to PTSD in a more general context would be misleading in critical care, for example, ‘difficulty concentratingʼ. Equally a PTSD tool would not capture important aspects of the critical care experience that may trigger distress, such as hallucinations or delusions. A further drawback is that PTSD measures are often completed with reference to an index event. In the critical care context it is unclear if this should be the event that resulted in them entering hospital, or the procedures they have endured within the hospital itself. In this complex situation it would be more appropriate to use a general measure of critical care distress.

Therefore, we developed a questionnaire to be used by critical care staff as a routine clinical tool with a dual purpose: to detect acute distress in critically ill patients, and also to predict patients who are at risk of future psychological morbidity. The main aim of this study was to collect data to analyse the reliability, validity and other psychometric properties of the questionnaire, named the intensive care psychological assessment tool (IPAT). Our hypotheses were that the IPAT would form a uni-dimensional scale of critical-care-related distress, with internal reliability ≥8 and test-retest reliability ≥8. We hypothesised that the IPAT total score would be significantly associated with concurrent anxiety, depression and general distress, and with future PTSD, depression, anxiety or general psychological morbidity at 3 months. The IPAT would have at least 75% sensitivity and 75% specificity to detect both current distress and future psychological morbidity.

## Materials and methods

### Development of the IPAT

The IPAT was adapted from the 18-item intensive care stress scale (ICUSS), previously created as a research tool for a prospective cohort study of intensive care psychological outcomes [2]. The ICUSS was derived from a comprehensive literature review of stressors and stress reactions in intensive care, and had four sub-scales: physical stress, delirious symptoms, control and support. Previous work showed that the ICUSS has good reliability, and concurrent and predictive validity [2].

To develop the IPAT, we added four mood-items to the ICUSS (the cohort study demonstrated that mood disturbance in critical care was one of the strongest risk factors for future psychological morbidity) and then shortened the resulting IPAT to fourteen items - communication, difficulty breathing, pain, sleep, anxiety, panic, depression, disorientation, delusions, hallucinations, intrusive memories, amnesia and self-reported psychological history. Items from ICUSS were selected for IPAT if they had significant medium to large correlation with ICUSS or mood scales and with at least one outcome (self-reported PTSD, depression and anxiety) in the cohort study, and satisfied NICE CG83 requirements [13]. Correlations of items selected for IPAT and concurrent stress or mood scores in the previous cohort study [2] ranged from *r* =0.42, *P* <0.01 to *r* =0.75, *P* <0.01. Correlation of items selected for IPAT and psychological outcomes in the cohort study ranged from *r* =0.25, *P* <0.01 to *r* =0.47, *P* <0.01. There are three possible responses for each item (no; yes a bit; yes a lot). Patients are asked to respond about feelings ‘since you’ve been in intensive care’. Data from the cohort study were useful to guide development of the IPAT, but further data were needed to validate it.

### Validation of the IPAT

#### Participants

##### Inclusion criteria

The inclusion criteria were that patients had been at least 48 hours in the critical care unit; had received level-two care (single organ support, as defined by The Intensive Care Society (ICS), UK) [14] or level-three care (mechanical ventilation >24 h, or support for two or more organs, ICS, UK); were English-speaking and able to communicate by an intelligible method; were awake and alert at the time of answering the questionnaire (for example, Glasgow coma score (GCS) of 15); were >18 years of age.

##### Exclusion criteria

Patients were excluded if they had continuing confusion, or GCS <15 (up to time of discharge from unit); were delirious at the time of screening (previous delirium was not an exclusion); had serious neurological impairment.

#### Setting

The setting was the critical care unit, a general surgical/medical 35-bedded unit, at University College Hospital, London.

#### Ethics

The study was approved by the Joint University College London/University College London Hospitals Committee on the Ethics of Human Research. Reference: 08/H0715/75. Eligible patients were asked for their informed consent before taking part in the study.

#### Procedure

Day 1 was defined as the first day that patients were un-sedated, awake and alert (GCS 15), potentially able to answer a questionnaire, and therefore in a mental state that indicated capacity to provide informed consent. Day 2 was a day later. Patients who were currently delirious were not approached. However, once the delirium stopped, they were approached and invited to join the study. On day 1, patients were administered the 14-item IPAT, usually by a bedside nurse, or occasionally by a member of the research team. Later on day 1, patients were administered a validation questionnaire comprising a short 6-item state anxiety scale from the Spielberger state-trait anxiety inventory (STAI) [15]; the patient health questionnaire-2 (PHQ-2) [16]; and the physical stress subscale of the condensed memorial symptom assessment scale (CMSAS) adapted for this study [17].

Some of the sample (56 patients) were administered the IPAT again on day 2 to allow the estimation of test-retest reliability. Three months after discharge from critical care, patients were posted a follow-up questionnaire comprising the post-traumatic stress disorder diagnostic scale (PDS), a validated scale for PTSD symptoms [18], the Center for Epidemiologic Studies depression scale (CES-D) for depression symptoms [19], and a short form of the STAI for anxiety [20]. The PDS items were adapted to refer specifically to the intensive care experience, as PDS authors have advised items be answered in relation to a specific trauma.

The protocol for the validation study included the opportunity to suspend the study after the first 15 patients if any aspects of the study or tool were problematic. Nurses administering the IPAT to the first 15 participants were asked to fill in a simple 8-item questionnaire, including 4 items for patients, after they had completed the IPAT, to assess the feasibility, interpretability and acceptability of the IPAT (see Additional file 1). If problems were identified by this process, the protocol would be revised, and data from the 15 patients would not be included in the study. However if no problems were identified, the data from the first 15 participants would be included in the study.

#### Socio-demographic and clinical data collection

Other data were collected from electronic patient notes: age, gender and clinical data; acute physiology and chronic health evaluation II (APACHE II) score [21]; maximum level of care received (2 or 3); length of stay in the critical care unit; number of critical care admissions; type of admission (surgical/non-surgical); diagnosis; past medical history; past psychological history; type and number of organs supported; level of sedation; drugs with potential psychoactive effects given; and CAM-ICU test results [22].

#### Assessment of psychometric properties

##### Scale analysis

IPAT items forming a consistent scale were selected using non-parametric item-response modelling (Mokken analysis).

##### Reliability

Test-retest reliability of the IPAT was estimated for a subset of patients who were administered the questionnaire twice. Internal consistency was estimated with Cronbach’s alpha.

##### Validity

Face validity of IPAT items was assessed by asking three nurses, three doctors and three patients who were formerly treated in the critical care unit, if they thought each item of the IPAT reflected a given aspect of patient experience. Responses were yes/no, with space for further comment.

For concurrent criterion validity, although there is no gold standard for measuring critical-care-related distress, we identified criterion measures for each aspect of the IPAT. Anxiety items of IPAT should correlate with the STAI, low-mood items of IPAT should correlate with the PHQ-2, and physical stress items should correlate with the CMSAS physical stress scale. IPAT delirium scores (based on recall of delirious symptoms such as hallucinations earlier in the critical care admission) should correlate with an ‘ever/never’ delirious factor based on patients’ earlier CAM-ICU results (the CAM-ICU is routinely carried out in this critical care unit). During the study it was found that CAM-ICU testing was not done consistently due to staff turnover and a new computer system being installed. Therefore, other indications of delirium from the notes were recorded and combined with CAM-ICU results for a further variable ‘likely delirium’. We hypothesised that the total IPAT score should also correlate with the STAI score, the PHQ-2, general distress (either anxiety or depression) and CAM-ICU. All correlations are reported as Pearson or Spearman’s (for non-parametric data) correlation coefficients.

For predictive validity, IPAT scores should correlate with scores on validated PTSD, depression and anxiety questionnaires and general psychological morbidity (above the cut-point for either PTSD, depression or anxiety) at three months.

### Sample size calculation (predictive validity)

There are no definitive criteria for the required sample size in a validation study of this kind, as the properties of the items and their scalability are not known in advance. However, assuming a median inter-item correlation of around 0.3, and that items are reasonably well separated, a sample size of 100 patients is sufficient for the proposed analysis. As 3-month data were collected to test for predictive validity, a sample size of 150 was proposed to allow for a one-third loss to follow up.

### Sample size calculation (test-retest reliability)

The sample size requirement for test-retest reliability was based on the assumption that reliability would be around 0.8. For a sample size of 50 patients, the 95% confidence intervals of 0.70 to 0.87 allowed the rejection of the hypothesis that reliability was 0.8 if the observed reliability fell below 0.7 [23].

### Sensitivity and specificity analyses

Sensitivity and specificity were derived for concurrent anxiety, depression and general distress in the critical care unit, as well as future risk of self-reported PTSD, and general psychological morbidity using coordinates on the receiver operating characteristic (ROC) curve, and the best cut point on the IPAT scale identified.

## Results

### Recruitment and follow up of participants

**Figure 1:**
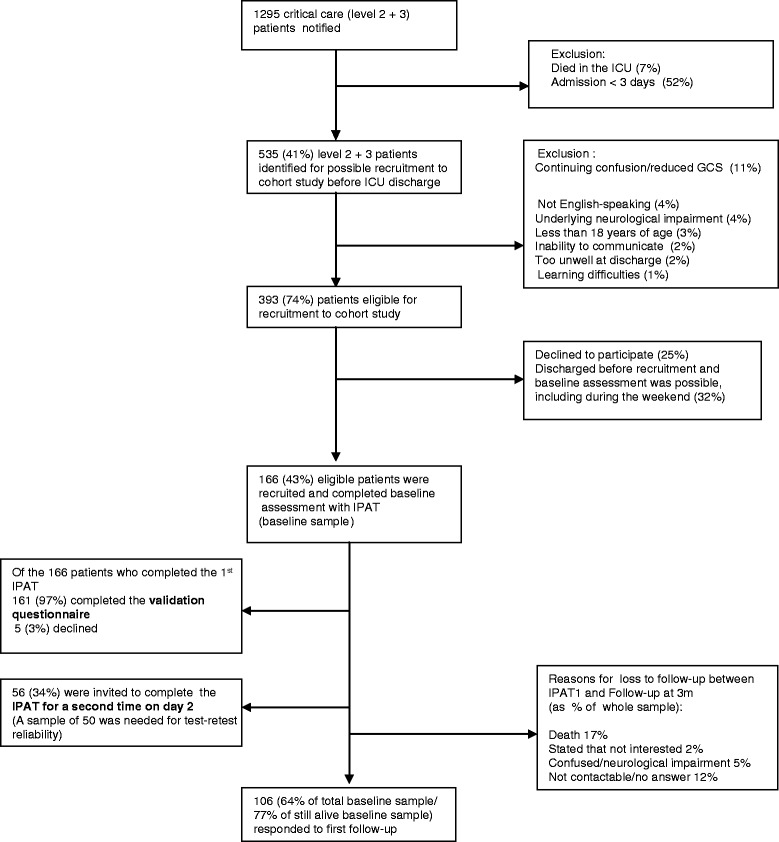
**Participant flow diagram, intensive care psychological assessment tool (IPAT) validation study.** A total of 166 patients (43% of eligible participants) were recruited into the study to validate the IPAT: 161 (97%) of the initial sample completed a validation questionnaire the same day, and 106 (77% of those still alive) completed a follow-up questionnaire on self-reported post-traumatic stress disorder, depression and anxiety at three months. Note: in the central column, the number in each box corresponds to a percentage of the number in the box above. The remaining percentage is accounted for in the exclusion boxes to the right. GCS, Glasgow coma score.

A sample of 166 patients was recruited (see Figure 1). After giving informed consent, the 166 patients completed IPAT once (day 1). The median time from critical care admission to assessment on day 1 was 6 days (IQR 7). Later on day 1, 161 (97%) of the patients completed the validation questionnaire. On day 2, 56 (34%) patients completed the IPAT a second time (a sample size of 50 was deemed sufficient for this purpose). Between the assessment in critical care and 3-month follow up, 28 (17%) patients died: 106 (77% of surviving patients) returned the follow-up questionnaire sent at 3 months.

### Patient characteristics and psychological scores

Table 1 shows there were no significant differences in any baseline characteristics or on IPAT scores between patients in day 1, day 2 and follow-up samples. Additional clinical data (not in Table 1) showed that more than half of the total sample was mechanically ventilated, and opiates and sedation were commonly administered. Regarding psychological outcomes, the mean score on the PDS was 10 (SD 9) and on the CES-D 17 (SD 13).

**Table 1:** **Sample characteristics for day 1, day 2 and follow up**

		**Day 1 sample**	**Day 2 sample**	**Follow-up sample**	***P*** **-values for differences between samples**
		**n =166**	**n =56**	**n =106**	
Age	Mean (SD)	57.6 (16)	58.5 (15.3)	58.5 (14.3)	*P* >0.05
Sex, male	Number (%)	93 (56%)	29 (52%)	58 (55%)	*P* >0.05
Psychological history^1^	Number (%)	44 (26%)	14 (25%)	28 (26%)	*P* >0.05
Type of admission					*P* >0.05
Surgical	Number (%)	68 (41%)	20 (35.7%)	48 (45.7%)	
Non-surgical	Number (%)	97 (59%)	36 (64%)	57 (54%)	
APACHE II score	Mean (SD)	19.6 (6.5)	21.1 (7)	18.8 (6.2)	*P* >0.05
Highest level of care					*P* >0.05
Level 2	Number (%)	61 (37%)	20 (36%)	44 (42%)	
Level 3	Number (%)	104 (63%)	36 (64%)	61 (58%)	
Length of stay in critical care unit	Median (IQR)	9 (13)	11 (30)	8 (8)	*P* >0.05
Days of sedation	Mean (SD)	3.2 (5.8)	4.5 (7.9)	2.7 (4.9)	*P* >0.05
Day IPAT first given	Median (IQR)	6 (7)	6 (12)	5 (7)	*P* >0.05
IPAT day-1 score	Mean (SD)	7.4 (4.7)	7.4 (4.7)	7.8 (4.7)	*P* >0.05
Mortality at 3 months	Number (%)	28 (17%)	9 (16%)	n/a^2^	*P* >0.05

^1^Documented history of psychological problems, including depression, anxiety, alcohol dependence, substance abuse, or combinations of these. ^2^Mortality reflects those who died by the 3-month follow-up point. All those in the follow-up sample had by definition survived to that point. APACHE II, acute physiology and chronic health evaluation II.

### Feasibility questionnaire (Additional file 1)

#### Results

All patients finished the IPAT; the layout was clear to all nurses; instructions were clear to all nurses and patients; no patients found the questions difficult to answer; questions were clear and unambiguous to 14 patients (one patient found an item confusing but could not remember which); 14 patients did not object to answering any questions.

#### Time taken to complete IPAT

Nine patients completed in 5 minutes or less; three in 10 minutes; two in 20 minutes; one in 40 minutes (throughout the whole study a minority of patients wanted to discuss their responses in more depth with their nurse).

### Item selection

Non-parametric Mokken-scale analysis (see Table 2) selected 10 of the 14 IPAT items as the components of a scale with reliability >0.8 (good). The ten items were hopelessness, tension, panic, delusions, intrusive memories, sadness, sleeplessness, communication difficulties, hallucinations and disorientation. The four excluded items were pain, difficulty breathing, amnesia for intensive care stay, and self-reported psychological history. This scale structure was also seen with day-2 IPAT data (reliability =0.8). All further analyses were carried out using the 10-item version of IPAT.

**Table 2:** **Mokken scaling (non-parametric factor analysis) of the intensive care psychological assessment tool (IPAT) (day 1) demonstrating that 10 of 14 original items form a scale**

**IPAT items forming scale**	**IPAT questions**	***H***
IPAT8	Have you been feeling hopeless?	0.59
IPAT5	Have you been feeling tense?	0.59
IPAT7	Have you been feeling panicky?	0.55
IPAT11	Have you felt that people were *deliberately* trying to harm or hurt you?	0.52
IPAT12	Do upsetting memories of intensive care keep coming into your mind?	0.49
IPAT6	Have you been feeling sad?	0.46
IPAT4	Has it been difficult to sleep?	0.44
IPAT1	Has it been hard to communicate?	0.41
IPAT10	Have you had hallucinations (seen or heard things you suspect were not really there)?	0.38
IPAT9	Have you felt disorientated (not quite sure where you are)?	0.37

*H*, Loevinger’s coefficient. The remaining four items had *H* <0.30, and were therefore excluded.

### Reliability

The correlation of IPAT total day-1 and IPAT total day-2 scores showed that test-retest reliability was 0.8. For the internal reliability for IPAT day-1 scores, Cronbach alpha = 0.8, and for IPAT day-2 scores, Cronbach alpha = 0.8.

### Face validity

Three doctors, three nurses and three patients returned a face-validity questionnaire. The IPAT scored 89% for face validity (nine people rated 10 items each; 80 out of 90 ratings were positive). The other 10 ratings were mostly ‘not sureʼ or suggestions for slight alterations to wording, rather than answering no. One patient, one nurse and one doctor indicated that all items had clear face validity. One patient, one nurse and one doctor indicated that 9 out of 10 items had clear face validity. Two patients, one nurse and one doctor indicated 8 items had clear face validity.

### Concurrent validity

Table 3 shows that there were large significant correlations between IPAT anxiety and STAI anxiety scores measured on the same day, between IPAT depression and PHQ-2 depression scores the same day, and between total IPAT, STAI and PHQ-2 scores on the same day. There was medium significant correlation between previous CAM-ICU status and both IPAT total and IPAT delirious-symptom scores. Correlation was slightly increased when ‘likely deliriumʼ (combining additional observations of delirium with CAM-ICU results) was used.

**Table 3:** **Concurrent and criterion validity: correlation between intensive care psychological assessment tool (IPAT) scores, concurrent anxiety and depression measures, and previously assessed delirium**

	**Validating anxiety scale**	**Validating depression scale**	**Validating delirium test (ever/never)**	**Likely delirium**
IPAT total score	0.7**	0.62**	0.34**	0.37**
IPAT anxiety score	0.69**			
IPAT depression score		0.58**		
IPAT delirious symptoms score			0.28**	0.29**

Validating anxiety scale, state trait anxiety inventor short form [15], usually administered day 1; validating depression scale, patient health questionnaire-2 [16], day 1. Validating delirium test, confusion assessment method for the intensive care unit (CAM-ICU) [22] (tested earlier in admission); IPAT anxiety, tense + panic items; IPAT depression, sad + hopeless; IPAT delirious symptoms, disorientated + hallucination + delusion. Likely delirium includes other indications of delirium from notes, with CAM-ICU results.**P <0.01.

### Sensitivity and specificity of IPAT as a measure of acute distress in critical care

With a cut point ≥7, the IPAT (day-1 data) had a sensitivity of 82% (95% CI 70, 91) and specificity of 65% (95% CI 55, 75) to detect concurrent anxiety in critical care patients. With the same cut point, the IPAT had 80% sensitivity (95% CI 68, 89) and 66% specificity (95% CI 55, 75) to detect concurrent low mood in critical care patients; and 75% sensitivity (95% CI 65, 84) and 74% specificity (95% CI 62, 83) to detect acute distress in critical care patients. The area under the curve (AUC) was 0.8 in all three cases.

### Predictive validity

In Table 4 we can see that there were highly significant correlations between IPAT total scores and the psychological outcomes of PTSD, depression and anxiety symptoms at 3 months. By Fisher’s *z*-transformation, none of the correlations between IPAT and other measures differed significantly between day 1 and day 2 (*P* >0.05). No significant relationships were found between CAM-ICU delirium results and psychological outcomes.

**Table 4:** **Predictive validity: correlation of intensive care psychological assessment tool (IPAT) and other scores with outcomes measured at 3 months**

	**PTSD**	**Depression**	**Anxiety**
	**at 3 months**	**at 3 months**	**at 3 months**
IPAT1 total, (day1, n =166)	0.4**	0.34**	0.25*
IPAT2 total (day 2, n =56)	0.64**	0.55**	0.45*
Validating anxiety scale (STAI) [15]	0.4**	0.29**	0.33**
Validating depression scale (PHQ-2) [16]	0.33**	0.28**	0.29**
Validating delirium test (CAM-ICU) [22]	0	0.17	0.12

*P <0.05; ** *P* <0.01. PTSD, post-traumatic stress diagnostic scale (PDS) [18]; Depression at 3 m, Centre for Epidemiological Studies depression scale (CES-D) [19]; Anxiety, short form of the state trait anxiety inventory (STAI) [20]; PHQ-2: patient health questionnaire-2 [16]; CAM-ICU, confusion assessment method in ICU [22].

### Sensitivity and specificity of IPAT as a predictor of PTSD and general psychological morbidity at 3 months

With a cut point ≥7, the IPAT (day-1 data) had 71% sensitivity (95% CI 49, 87) and 48% specificity (95% CI 37, 59) for future diagnosis of PTSD (AUC =0.6). With a cut point ≥7, the IPAT had 69% specificity (95% CI 55, 82) and 57% sensitivity (95% CI 43, 70) to predict future psychological morbidity (AUC =0.7).

## Discussion

The IPAT was developed as a quick, simple screening tool for routine use by critical care staff to detect acute distress in critically ill patients, as well as their risk of future psychological morbidity. Both are well-documented problems in critical care [1-4], yet no validated easy-to-use screening tool was available for this purpose [7]. The IPAT was developed in line with the requirements of the UK NICE guideline CG83, that all critical care patients should be psychologically assessed, so that psychological support can be offered to patients, with the aim of reducing acute distress and future psychological morbidity [7].

The validation study carried out with 166 patients in our critical care unit confirmed that 10 of 14 original items formed a reliable unitary scale measuring psychological distress in critical care. The study showed that the IPAT was feasible and acceptable, being quick and easy for both patients and nurses to use and understand. Further analysis demonstrated that the IPAT has good test-retest reliability, internal consistency, face validity, construct validity, concurrent validity and predictive validity.

Based on our hypotheses on reliability, sensitivity and specificity, the IPAT was accepted as a screening tool to detect acute distress in the critical care unit, subject to replication and further validation. However further work would be needed to refine the psychometric properties of the IPAT, if it were also to be used as a tool to predict future psychological morbidity.

The IPAT shows promise as a screening tool to detect acute distress in critical care patients once they are awake, alert and orientated. Staff should be adequately trained to provide extra psychological support to patients identified as suffering distress in the critical care unit, and to document concerns about patients’ psychological state, to be handed over when patients are transferred to other wards. Studies are currently being carried out to evaluate psychological interventions started in critical care units, and continued on general wards.

Limitations to the study include the issue that criterion validation of delirious-symptom items (for example, hallucinations) in the IPAT was hampered because the CAM-ICU was not carried out consistently during the study period, due to staffing turnover and the introduction of a new computer system. Outcomes of the study (PTSD, depression and anxiety symptoms) were measured using self-report questionnaires, rather than gold-standard clinical interviews and specifically trained interviewers. The participation rate of eligible patients in the study (43%) was low and a potential source of bias. A further limitation was the variable time of administration of the IPAT: this potentially influenced the patients’ scores, as intervening events could not be eliminated or controlled for.

## Conclusions

Our study to validate the IPAT showed that it had good reliability, concurrent validity, predictive validity, sensitivity and specificity to detect acute distress in critical care. Based on the present findings, the IPAT could provide a way of allowing critical care staff to assess psychological distress among critical care patients after replication and further validation. Further work is also planned to establish the psychometric properties of the IPAT as a tool to predict the risk of future psychological morbidity, including PTSD, in critical care patients.

## Key messages

- Patients often experience acute psychological stress in critical care units, and suffer from psychological morbidity in the months following their critical care admission
- Conducting routine psychological assessments of critical care patients is considered best practice, but to date no quick, simple, critical care-specific tool for staff to use for this purpose has been developed and validated
- The IPAT was developed at University College Hospital, London, as a screening tool to improve critical care staff’s detection of acute psychological stress and risk of future psychological morbidity in critical care patients. Our validation study showed that the IPAT had good psychometric properties, including reliability, concurrent and predictive validity
- We recommend that the IPAT should be considered for routine clinical use to detect acute distress among critical care patients who are alert, awake and orientated, after replication and further validation
- Further work is needed to validate the IPAT as a potential tool to predict future psychological morbidity

## Additional file

Additional file 1
**Feasibility questionnaire completed by nurses administering the intensive care psychological assessment tool (IPAT).**

## Abbreviations

APACHE: acute physiology and chronic health evaluation
AUC: area under the curve
CAM-ICU: confusion assessment method for the intensive care unit
CES-D: Center for Epidemiologic Studies depression scale
CMSAS: condensed memorial symptom assessment scale
GCS: Glasgow coma score
ICUSS: intensive care stress scale
IPAT: intensive care psychological assessment tool
NICE: National Institute for Health and Care (previously Clinical) Excellence
PDS: post-traumatic stress disorder diagnostic scale
PHQ-2: patient health questionnaire-2
PTSD: post-traumatic stress disorder
ROC: receiver operating characteristic
STAI: state trait anxiety inventory
UCH: University College Hospital
UCL: University College London
UCLH: University College London Hospitals
