# Supplementary material for: Detecting acute distress and risk of future psychological morbidity in critically ill patients: validation of the intensive care psychological assessment tool
Source: Crit Care. 2014 Sep 24;18(5):519. doi: 10.1186/s13054-014-0519-8 (PMC4207352; doi:10.1186/s13054-014-0519-8)
Supplement: Additional file 1 — Feasibility questionnaire completed by nurses administering the intensive care psychological assessment tool (IPAT). [file 13054_2014_519_MOESM1_ESM.doc]

**IPAT Feasibility Questionnaire**

1. Did the patient manage to finish the interview? **Yes/No** (please circle). If no, suggest reasons why not.

___________________________________________________________

2. If yes, how long did it take you to complete the interview with the patient?

___________________________________________________________

2. Was the layout clear to you? **Yes/No** (please circle).

If not, please give details.

__________________________________________________________

3. Were the instructions clear to you? **Yes/No** (please circle).

If not, please give details.

___________________________________________________________

4. Ask the patient if the instructions were clear to them. **Yes/No** (please circle). If not, please give details.

___________________________________________________________

5. Ask the patient if they found any questions unclear or ambiguous. **Yes/No** (please circle). If yes, please give details.

___________________________________________________________

5. Ask the patient if any questions were difficult to answer. **Yes/No** (please circle). If yes, please give details.

___________________________________________________________

6. Ask the patient if they objected to answering any questions? **Yes/No** (please circle). If yes, please give details.

___________________________________________________________

7. Do you have any other comments?
